# Interaction between Mesocortical and Mesothalamic Catecholaminergic Transmissions Associated with NMDA Receptor in the Locus Coeruleus

**DOI:** 10.3390/biom10070990

**Published:** 2020-07-01

**Authors:** Motohiro Okada, Kouji Fukuyama

**Affiliations:** Department of Neuropsychiatry, Division of Neuroscience, Graduate School of Medicine, Mie University, Tsu 514-8507, Japan; k-fukuyama@clin.medic.mie-u.ac.jp

**Keywords:** N-methyl-D-aspartate, schizophrenia, mood disorder, L-glutamate, GABA, catecholamine

## Abstract

Noncompetitive N-methyl-D-aspartate/glutamate receptor (NMDAR) antagonists contribute to the pathophysiology of schizophrenia and mood disorders but improve monoaminergic antidepressant-resistant mood disorder and suicidal ideation. The mechanisms of the double-edged sword clinical action of NMDAR antagonists remained to be clarified. The present study determined the interaction between the NMDAR antagonist (MK801), α1 adrenoceptor antagonist (prazosin), and α2A adrenoceptor agonist (guanfacine) on mesocortical and mesothalamic catecholaminergic transmission, and thalamocortical glutamatergic transmission using multiprobe microdialysis. The inhibition of NMDAR in the locus coeruleus (LC) by local MK801 administration enhanced both the mesocortical noradrenergic and catecholaminergic coreleasing (norepinephrine and dopamine) transmissions. The mesothalamic noradrenergic transmission was also enhanced by local MK801 administration in the LC. These mesocortical and mesothalamic transmissions were activated by intra-LC disinhibition of transmission of γ-aminobutyric acid (GABA) via NMDAR inhibition. Contrastingly, activated mesothalamic noradrenergic transmission by MK801 enhanced intrathalamic GABAergic inhibition via the α1 adrenoceptor, resulting in the suppression of thalamocortical glutamatergic transmission. The thalamocortical glutamatergic terminal stimulated the presynaptically mesocortical catecholaminergic coreleasing terminal in the superficial cortical layers, but did not have contact with the mesocortical selective noradrenergic terminal (which projected terminals to deeper cortical layers). Furthermore, the α2A adrenoceptor suppressed the mesocortical and mesothalamic noradrenergic transmissions somatodendritically in the LC and presynaptically/somatodendritically in the reticular thalamic nucleus (RTN). These discrepancies between the noradrenergic and catecholaminergic transmissions in the mesocortical and mesothalamic pathways probably constitute the double-edged sword clinical action of noncompetitive NMDAR antagonists.

## 1. Introduction

Recently, the impact of N-methyl-D-aspartate/glutamate receptor (NMDAR) modulation has been a fundamental psychopharmacological discussion, since (S)-ketamine was approved by the Food and Drug Administration (FDA) in early 2019 as a rapid-acting antidepressant for the treatment of major depressive disorders [1]. In particular, monoaminergic antidepressants exhibit limited effectiveness against the suicidal ideation of mood disorders, whereas ketamine has also been shown to have distinct and independent anti-suicidal effects in patients with mood disorders [2,3]. Therefore, ketamine can improve monoaminergic antidepressant-resistant cognitive dysfunction in mood disorders. Contrary to these clinical advantages of ketamine, numerous clinical and preclinical studies have reported that noncompetitive NMDAR antagonists, such as phencyclidine, ketamine, and dizocilpine (MK801), contribute to the pathophysiology of schizophrenia, as NMDAR antagonists generate schizophrenia-like positive–negative symptoms and cognitive impairments in healthy individuals and experimental animal models, as well the exacerbation of psychotic symptoms of patients with schizophrenia [4,5,6,7,8,9,10,11,12,13].

Acute ketamine administration rapidly improves several symptoms in mood disorders, but reduces attention [14,15]. It has been considered that noradrenergic transmission associated with the locus coeruleus (LC) is one of the major fundamental neural circuits for the regulation of attention [16]. It has been well known that the major pharmacological mechanism of ketamine is the inhibition of NMDAR, whereas various other target systems have been explored in order to understand the paradoxical clinical actions of ketamine, including monoaminergic transmission [17,18,19]. The inhibition of the transporters of serotonin and norepinephrine provides an anti-depressive action and the improvement of dysfunctions of cognition and emotional perception in patients with major depression [20,21]. Noradrenergic signalling is also considered to be a candidate target of the mechanism of action of ketamine. Ketamine dose-dependently increases norepinephrine release in the medial prefrontal cortex, but clonidine inhibits ketamine-induced norepinephrine release via the activation of the presynaptic α2 adrenoceptor [22,23]. These findings suggest that ketamine enhances noradrenergic transmission similar to serotonin/norepinephrine transporter inhibitors. Contrary to microdialysis studies, norepinephrine transporter inhibiting antidepressants and ketamine suppress and enhance neuronal activities in the LC, respectively [24,25]. These contradictive demonstrations between electrophysiological and microdialysis studies suggest that the LC is probably one of the major target regions of the clinical action of ketamine, but the norepinephrine transporter inhibition probably cannot play an important role in the stimulatory effects of ketamine on noradrenergic transmission.

Recently, we reported that the enhanced glutamatergic transmission in the thalamocortical pathway is one of the candidate responsible pathways of systemic NMDAR antagonist induced L-glutamate release in the frontal cortex [26,27,28,29,30,31]. The major thalamocortical glutamatergic pathways [32] composed of projection form the mediodorsal thalamic nucleus (MDTN) to the medial prefrontal cortex, insula, and orbitofrontal cortex (OFC) [26,27,30,31,33,34,35,36,37,38,39]. These thalamocortical glutamatergic pathways contribute to the constitution of cognition, and to stability and flexibility against environmental changes [26,27,28,29,30,31,33,34,40,41]. In particular, mesothalamic noradrenergic transmission plays important roles in the appropriate response of thalamocortical glutamatergic transmission to various inputs [36]. The activation of the α2A adrenoceptor in the mesothalamic noradrenergic pathway enhances intrathalamic inhibition of γ-aminobutyric acid (GABA) [36], resulting in the attenuation of thalamocortical glutamatergic transmission [26,27,30,31,36]. A clinical study using functional magnetic resonance imaging reported that a subanaesthetic dose of ketamine injection reduced connectivity between the LC and MDTN [42], consistent with the hypothesis that ketamine augments noradrenergic transmission from the LC to various target regions.

LC projects selective noradrenergic and catecholamine coreleasing (norepinephrine and dopamine) terminals to the frontal cortex (mesocortical pathways) and noradrenergic terminal to the reticular thalamic nucleus (RTN) (mesothalamic pathway) [36,37,38,43]. The thalamocortical glutamatergic pathway receives GABAergic inhibition from the RTN, and projects terminals to superficial layers in the frontal cortex [27,29,31]. The thalamocortical glutamatergic terminal stimulates mesocortical catecholaminergic coreleasing terminals in the superficial layers of the frontal cortex via α-amino-3-hydroxy-5-methyl-4-isoxazolepropionic acid (AMPA)/glutamate receptor (AMPAR), but does not make contact with the mesocortical selective noradrenergic terminals [30,31,37,38]. These clinical and preclinical findings indicate that the interaction between the mesocortical and mesothalamic noradrenergic/catecholaminergic transmissions on thalamocortical glutamatergic transmission contributes to the double-edged sword clinical action of the NMDAR antagonist on mood and cognition. Therefore, according to our hypothesis, the present study determines the effects of MK801, α1 adrenoceptor antagonist (prazosin), and α2A adrenoceptor agonist (guanfacine) on mesocortical and mesothalamic noradrenergic/catecholaminergic transmissions and thalamocortical glutamatergic transmission using multiprobe microdialysis.

## 2. Materials and Methods

### 2.1. Experimental Animals

The animal care and experimental procedures in this report were performed in compliance with the ethical guidelines established by the Institutional Animal Care and Use Committee at Mie University (no. 29-22-R2). All of the studies involving animals were reported in accordance with the relevant ARRIVE guidelines [44]. A total of 92 male Sprague-Dawley rats (approximately 250 g, 7−8 weeks old, SLC, Shizuoka, Japan) were used in this study, and were maintained in a controlled environment (22 °C ± 1 °C) with a 12-h light/12-h dark cycle.

### 2.2. Chemical Agents

Dizocilpine (MK801; noncompetitive NMDAR antagonist), muscimol (MUS; GABA_A_ receptor agonist), and prazosin (PRZ; α1 adrenoceptor antagonist) was obtained from Fujifilm-Wako (Osaka, Japan). Guanfacine (GUNA; α2A adrenoceptor agonist) and α-amino-3-hydroxy-5-methyl-4-isoxazolepropionic acid (AMPA; AMPAR agonist) were obtained from Cosmo Bio (Tokyo, Japan). Each compound was prepared on the day of the experiments. These drugs were perfused in modified Ringer’s solution (MRS) containing 145 mM Na^+^, 2.7 mM K^+^, 1.2 mM Ca^2+^, 1.0 mM Mg^2+^, and 154.4 mM Cl^−^, which was adjusted to pH 7.4 using 2 mM phosphate buffer and 1.1 mM Tris buffer [30,31]. MK801, GUNA, MUS, and AMPA were dissolved in MRS directly. PRZ was initially dissolved at a concentration of 25 mM in dimethyl sulfoxide.

According to previous reports, in the present study, to study the effects of MK801, MUS, PRZ, and GUNA, each rat was administered with MK801 (1 and 5 μM) [26,27,29,30,31], MUS (1 μM) [37,38], AMPA (100 μM) [30,31,33,36], PRZ (100 μM) [43], and GUNA (0.2 μM) [36] into the LC, MDTN or RTN.

### 2.3. Microdialysis System

The rats were anesthetized with 1.8% isoflurane and were then placed in a stereotaxic frame for the implantation of dialysis probes [38,45]. Concentric direct insertion type dialysis probes (0.22 mm diameter; Eicom, Kyoto, Japan) were implanted into the orbitofrontal cortex (OFC; A = +3.2 mm, L = +2.4 mm, V = −6.5 mm, relative to bregma; 0.22 mm diameter, 2 mm exposed membrane; Eicom), mediodorsal thalamic nucleus (MDTN; A = −3.0 mm, L = +0.6 mm, V = −6.4 mm, relative to bregma; 0.22 mm diameter, 2 mm exposed membrane; Eicom) at a lateral angle of 30°, reticular thalamic nucleus (RTN: A = −1.4 mm, L = +1.2 mm, V = −7.2 mm, relative to bregma) at a lateral angle of 30°, or locus coeruleus (LC: A = −9.7 mm, L = +1.3 mm, V = −7.6 mm, relative to bregma; 0.22 mm diameter, 1 mm exposed membrane; Eicom) [31,34,36,40,41,46].

During recovery and experimentation, the rats were housed individually in cages and were provided food and water ad libitum. The perfusion experiments were initiated 18-h after recovery from isoflurane anaesthesia [47,48,49]. During the experiments, single rats were placed in an in vivo microdialysis system for freely moving rats (Eicom, Kyoto, Japan), equipped with a two-channel swivel (TCS2-23; ALS, Tokyo, Japan). The perfusion rate was set at 2 μL/min in all of the experiments, using modified Ringer’s solution (MRS; composition described above) [47,50], and dialysates were collected over 20 min sampling epochs. The extracellular levels of norepinephrine, dopamine, L-glutamate, and GABA were measured 8 h after the start of the perfusions. After baseline recording, the perfusion medium was replaced with MRS containing MK801, PRZ, GUNA, or AMPA, as indicated. The dialysate samples were then injected into the ultra-high-performance liquid chromatography (UHPLC) apparatus. All of the samples were taken from freely moving animals.

After the microdialysis studies, the rat brains were removed following isoflurane overdose anesthesia. The locations of the dialysis probes were checked in each animal using histological examinations of 200 μm thick brain slices with a Vibratome 1000 (Technical Products International Inc., St. Louis, MO, USA).

### 2.4. Determination of Extracellular Levels of L-Glutamate, GABA, Dopamine and Norepinephrine

The extracellular levels of L-glutamate and GABA in the perfusate were analysed by UHPLC (xLC3185PU; Jasco, Tokyo, Japan) with fluorescence resonance energy transfer detection (xLC3120FP; Jasco) after dual derivatization with isobutyryl-l-cysteine and *o*-phthalaldehyde. The derivative reagent solutions were prepared by dissolving isobutyryl-l-cysteine (2 mg) or *o*-phthalaldehyde (1 mg) in 0.1 mL ethanol, followed by the addition of 0.9 mL of sodium borate buffer (0.2 M, pH 9.0) [30,31,47,50]. The automated precolumn derivation was performed by mixing 5 μL of sample, standard, or blank solutions with 5 μL of derivative reagent solution in reaction vials for 5 min before injection (xLC3059AS; Jasco). Derivative samples (5 μL) were injected using an autosampler (xLC3059AS; Jasco). The flow rate was set at 0.5 mL/min, and elution was performed using a linear gradient of mobile phases A (0.05 M acetate buffer, pH 5.0) and B (0.05 M acetate buffer containing 60% acetonitrile, pH 3.5) over 10 min [30,31,47,50]. The excitation and emission wavelengths of the fluorescence detector were set at 280 and 455 nm, respectively. The analytical column (YMC Triart C18, particle 1.8 μm, 50 × 2.1 mm; YMC, Kyoto, Japan) was maintained at 45 °C.

The concentrations of dopamine and norepinephrine were determined using UHPLC (xLC3185PU; Jasco) with an electrochemical detector (ECD-300; Eicom) by a graphite carbon electrode set at +450 mV (vs. an Ag/AgCl reference electrode) [30,31,47,50]. The analytical column (Triart C18, particle 1.8 μm, 30 × 2.1 mm; YMC) was maintained at 40 °C and the flow rate of the mobile phase was set at 400 μL/min. The mobile phase contained 0.1 M acetate buffer, 1% methanol, and 50 mg/L ethylenediaminetetraacetic acid (EDTA)-2Na (final pH 6.0) [51].

### 2.5. Data Analysis

To determine the extracellular transmitter levels, the sample order was set on the autosampler according to a random number table. The drug levels and sample sizes of each experimental group were selected according to previous studies [26,27,28,29,30,31,36]. All of the experiments in this study were designed with equivalent sized groups (n = 6), without carrying out a formal power analysis [26,27,28,29,30,31,36], and all of the data were expressed as mean ± standard deviation (SD), and *p* < 0.05 (two-tailed) was considered statistically significant. The drug concentration of the acute local administration (perfusion) was selected based on previous studies.

Levels of the transmitter were analysed by Mauchly’s sphericity test, followed by multivariate analysis of variance (MANOVA) (BellCurve for Excel v. 3.2, Social Survey Research Information Co., Ltd., Tokyo, Japan). If data did not violate the assumption of sphericity (*p* > 0.05), the F-value of MANOVA was analysed by sphericity assumed degrees of freedom. Contrary, if the assumption of sphericity was violated (*p* < 0.05), the F-value was analysed by Chi–Muller’s corrected degrees of freedom. When the F-value for the drug*time factors of MANOVA was significant, the data was analysed by Tukey’s post hoc test.

## 3. Results

### 3.1. Effects of NMDAR in the LC on Transmitter Release in the OFC, RTN and MDTN (Study_1)

To explore the threshold level of local administration (perfusion with) of MK801 into the LC, Study_1 was designed to determine the effects of perfusion with 1 μM and 5 μM MK801 into the LC on the extracellular levels of GABA in the LC and MDTN, and extracellular levels of norepinephrine and dopamine in the OFC and RTN (MK801-induced release). Additionally, to clarify the mechanisms of MK801-induced releases of norepinephrine, dopamine and GABA, the effects of perfusion with 100 μM PRZ (α1 adrenoceptor antagonist) or 0.2 μM GUNA (α2A adrenoceptor agonist) into the LC on MK801-induced releases of norepinephrine and dopamine in the OFC and RTN, and GABA release in the LC and MDTN were determined.

Perfusion mediums in the LC was commenced with MRS containing without (control) or with 100 μM PRZ or 0.2 μM GUNA. After the stabilisation of transmitter level in perfusate, perfusion medium in the LC was switched to the same MRS containing with MK801 (1 or 5 μM) for 180 min. Perfusion medium in the OFC, RTN and MDTN were maintained with MRS alone during Study_1.

#### 3.1.1. Effects of Local Administration of PRZ and GUNA into the LC on MK801-Induced Releases of Norepinephrine and Dopamine in the OFC

**Figure 1:**
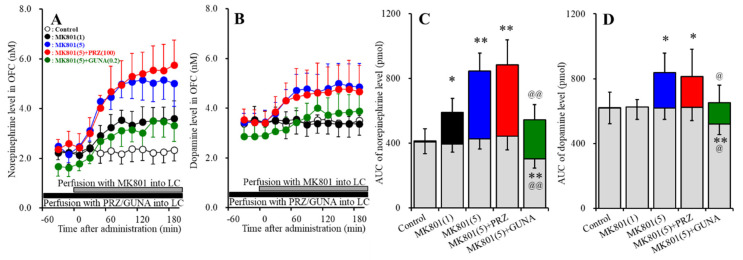
Effects of local administration of prazosin (PRZ) and guanfacine (GUNA) into the locus coeruleus (LC) on dizocilpine (MK801)-induced releases of norepinephrine (**A**) and dopamine (**B**) in the orbitofrontal cortex (OFC). Ordinates: mean ± standard deviation (SD) (n = 6) of extracellular catecholamine levels (nM); abscissa: time after MK801 administration (min). Black bars indicate perfusion with 100 μM prazosin (PRZ) or 0.2 μM guanfacine (GUNA) into the LC. Gray bars indicate perfusion with 1 μM or 5 μM MK801 into the LC. (**C**,**D**) indicate the area under curve (AUC) values of extracellular levels of norepinephrine and dopamine (pmol) after perfusion with MK801 (from 20 to 180 min), based on (**A**) and (**B**), respectively. Gray columns represent the AUC values before perfusion with MK801 (basal release). * *p* < 0.05, ** *p* < 0.01; relative to control, @ *p* < 0.05, @@ *p* < 0.01; relative to MK801 (5 μM) by MANOVA with Tukey’s post hoc test.

Perfusions with MK801 (1 and 5 μM) into the LC concentration-dependently increased extracellular norepinephrine level in the OFC [F_MK801_ (2,15) = 29.7 (*p* < 0.01), F_Time_ (4.0, 60.4) = 95.9 (*p* < 0.01), F_MK801*Time_ (8.1, 60.4) = 33.6 (*p* < 0.01)] (Figure 1A,C). Perfusion with 100 μM PRZ (α1 adrenoceptor antagonist) into the LC affected neither basal nor MK801-induced (5 μM MK801 into the LC) norepinephrine releases in the (Figure 1A,C). Perfusion with 0.2 μM GUNA (α2A adrenoceptor agonist) into the LC decreased both basal and MK801-induced norepinephrine release in the OFC [F_GUNA_ (2,15) = 29.3 (*p* < 0.01), F_Time_ (5.0, 75.0) = 104.1 (*p* < 0.01), F_GUNA*Time_ (10.0, 75.0) = 33.3 (*p* < 0.01)] (Figure 1A,C). Perfusions with MK801 (1 and 5 μM) into the LC concentration-dependently increased extracellular dopamine level in the OFC [F_MK801_ (2,15) = 9.0 (*p* < 0.01), F_Time_ (4.4, 65.4) = 8.8 (*p* < 0.01), F_MK801*Time_ (8.7, 65.4) = 11.4 (*p* < 0.01)] (Figure 1B,D). Perfusion with 5 μM MK801 into the LC increased extracellular dopamine level in the OFC, but 1 μM MK801 did not affect (Figure 1B,D). Perfusion with 100 μM PRZ into the LC affected neither basal nor MK801-induced dopamine release in the OFC (Figure 1B,D). Perfusion with 0.2 μM GUNA into the LC decreased both basal and MK801-induced dopamine release in the OFC [F_GUNA_ (2,15) = 6.2 (*p* < 0.05), F_Time_ (4.6, 68.8) = 25.6 (*p* < 0.01), F_GUNA*Time_ (9.2, 68.8) = 7.6 (*p* < 0.01)] (Figure 1B,D). Therefore, the threshold concentration of local administration of MK801 into the LC on releases of norepinephrine and dopamine in the OFC were lower than 1 μM and 5 μM, respectively. The noradrenergic transmission in LC-OFC is more sensitive to MK801 compared with that of dopamine.

#### 3.1.2. Effects of Local Administration of PRZ and GUNA into the LC on MK801-Induced Releases of Norepinephrine and Dopamine in the RTN

**Figure 2:**
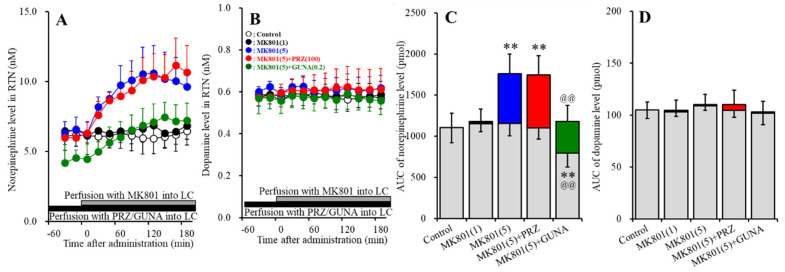
Effects of local administration of PRZ and GUNA into the LC on MK801-induced releases of norepinephrine (**A**) and dopamine (**B**) in the reticular thalamic nucleus (RTN). Ordinates: mean ± SD (n = 6) of extracellular catecholamine levels (nM); abscissa: time after MK801 administration (min). Black bars indicate perfusion with 100 μM PRZ or 0.2 μM GUNA into the LC. Gray bars indicate perfusion with 1 μM and 5 μM MK801 into the LC. (**C**,**D**) indicate the AUC values of extracellular levels of norepinephrine and dopamine (pmol) after perfusion with MK801 (from 20 to 180 min), based on (**A**) and (**B**), respectively. Gray columns represent the AUC values before perfusion with MK801 (basal release). ** *p* < 0.01; relative to control, and @@ *p* < 0.01; relative to MK801 (5 μM) by MANOVA with Tukey’s post hoc test.

Perfusions with MK801 (1 and 5 μM) into the LC concentration-dependently increased extracellular norepinephrine level in the RTN [F_MK801_ (2,15) = 17.7 (*p* < 0.01), F_Time_ (3.9, 58.7) = 16.4 (*p* < 0.01), F_MK801*Time_ (7.8, 58.7) = 14.2 (*p* < 0.01)] (Figure 2A,C). Perfusion with 5 μM MK801 into the LC increased extracellular norepinephrine level in the RTN, but 1 μM MK801 did not affect (Figure 2A,C). Perfusion with 100 μM PRZ into the LC affected neither basal nor MK801-induced norepinephrine release in the RTN (Figure 2A,C). Perfusion with 0.2 μM GUNA into the LC decreased both basal and MK801-induced norepinephrine release in the RTN [F_GUNA_ (2,15) = 16.4 (*p* < 0.01), F_Time_ (4.2, 62.9) = 43.8 (*p* < 0.01), F_GUNA*Time_ (8.4, 62.9) = 13.4 (*p* < 0.01)] (Figure 2A,C). Perfusions with MK801 (1 and 5 μM) into the LC did not affect the extracellular dopamine level in the RTN (Figure 2B,D). Neither perfusion with 100 μM PRZ nor 0.2 μM GUNA into the LC affected the extracellular dopamine level in the RTN (Figure 2B,D). Therefore, the threshold concentration of local administration of MK801 into the LC on norepinephrine release in the RTN was lower than 5 μM, but catecholaminergic neurones in the LC does not project to RTN.

#### 3.1.3. Effects of Local Administration of PRZ and GUNA into the LC on MK-801-Induced Reduction in GABA Release in the LC

**Figure 3:**
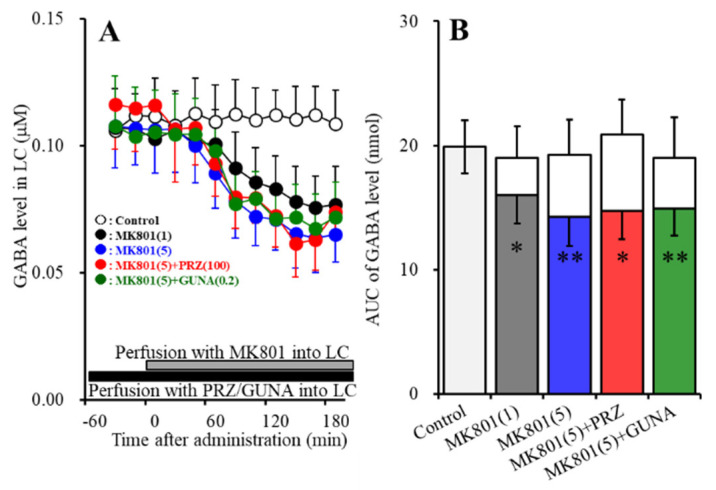
Effects of local administration of PRZ and GUNA into the LC on MK801-induced reduction in γ-aminobutyric acid (GABA) release in the LC. Ordinate: mean ± SD (n = 6) of extracellular GABA level (μM); abscissa: time after MK801 administration (min). Black bars indicate perfusion with 100 μM PRZ or 0.2 μM GUNA into the LC. Gray bars indicate perfusion with 1 μM and 5 μM MK801 into the LC. (**B**) indicates the AUC values of extracellular GABA level (nmol) after perfusion with MK801 (from 20 to 180 min), based on (**A**). Gray columns represent the AUC values before perfusion with MK801 (basal release). * *p* < 0.05, ** *p* < 0.01; relative to control by MANOVA with Tukey’s post hoc test.

Perfusions with MK801 (1 and 5 μM) into the LC concentration-dependently decreased extracellular GABA level in the LC [F_MK801_ (2,15) = 8.0 (*p* < 0.01), F_Time_ (6.3, 94.2) = 91.5 (*p* < 0.01), F_MK801*Time_ (12.6, 94.2) = 28.1 (*p* < 0.01)] (Figure 3A,B). Perfusion with 100 μM PRZ into the LC affected neither basal nor MK801-induced reduction in GABA release in the LC (Figure 3A,B). Perfusion with 0.2 μM GUNA into the LC also affected neither basal nor MK801-induced reduction in GABA release in the LC (Figure 3A,B).

#### 3.1.4. Effects of Local Administration of PRZ and GUNA into the LC on MK801-Induced GABA Release in the MDTN

**Figure 4:**
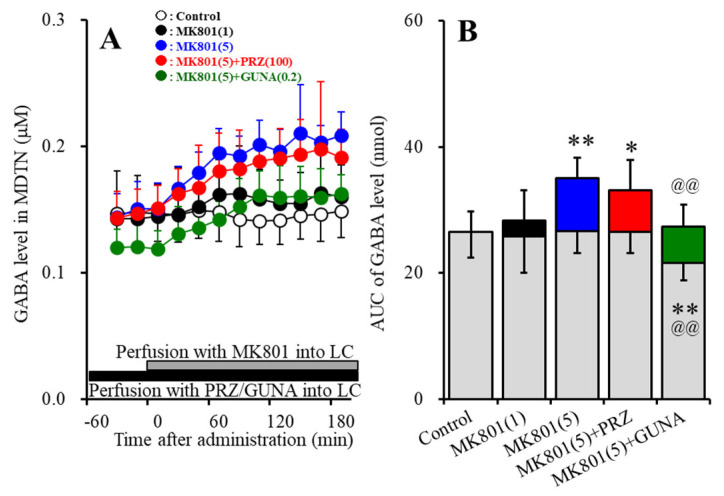
Effects of local administration of PRZ and GUNA into the LC on MK801-induced GABA release in the mediodorsal thalamic nucleus (MDTN). Ordinate: mean ± SD (n = 6) of extracellular GABA level (μM); abscissa: time after MK801 administration (min). Black bars indicate perfusion with 100 μM PRZ or 0.2 μM GUNA into the LC. Gray bars indicate perfusion with 1 μM and 5 μM MK801 into the LC. (**B**) indicates the AUC values of extracellular GABA level (nmol) after perfusion with MK801 (from 20 to 180 min), based on (**A**). Gray columns represent the AUC values before perfusion with MK801 (basal release). * *p* < 0.05, ** *p* < 0.01; relative to control, @@ *p* < 0.01; relative to MK801 (5 μM) by MANOVA with Tukey’s post hoc test.

Perfusions with MK801 (1 and 5 μM) into the LC concentration-dependently increased extracellular GABA level in the MDTN [F_MK801_ (2,15) = 6.7 (*p* < 0.01), F_Time_ (4.7, 70.1) = 14.7 (*p* < 0.01), F_MK801*Time_ (9.3, 70.1) = 8.2 (*p* < 0.01)] (Figure 4A,B). Perfusion with 5 μM MK801 into the LC increased extracellular GABA level in the MDTN, but 1 μM MK801 did not affect (Figure 4A,B). Perfusion with 100 μM PRZ into the LC affected neither basal nor MK801-induced GABA release in the MDTN (Figure 4A,B); however, perfusion with 0.2 μM GUNA into the LC decreased both basal and MK801-induced GABA release in the MDTN (Figure 4A,B).

### 3.2. Effects of Local Administration of MUS into the LC on MK801-Induced Releaes of Norepinephrine and Doapmine in the OFC and RTN (Study_2)

Study_1 suggests that MK801-induced releases of dopamine in the OFC and norepinephrine in the OFC and RTN are possibly generated by intra-LC GABAergic disinhibition, similar to serotonergic and glutamatergic transmission [29,31,34]. Based on the previous demonstration, to explore the mechanisms of MK801-induced catecholamine release in the OFC and RTN, the effects of perfusion with 1 μM MUS (GABA_A_ receptor agonist) into the LC on MK801-induced releases of norepinephrine in the OFC, RTN and dopamine in the OFC were determined [37,38,48,52]. Perfusion mediums in the OFC and RTN were maintained with MRS alone during Study_2. Perfusion mediums in the LC was commenced with MRS containing without (control) or with 1 μM MUS. After the stabilisation of transmitter level in perfusate, perfusion medium in the LC was switched to the same MRS containing with 5 μM MK801 for 180 min.

**Figure 5:**
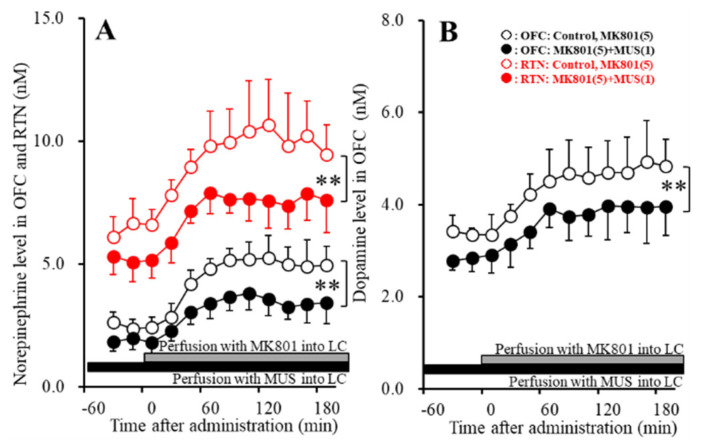
Effects of local administration of muscimol (MUS: 1 μM) into the LC on MK801-induced releases of norepinephrine in the OFC and RTN (**A**) and dopamine in the OFC (**B**). Ordinate: mean ± SD (n = 6) of extracellular levels of norepinephrine and dopamine (nM); abscissa: time after MK801 administration (min). Closed and gray bars indicate perfusion with 1 μM MUS and 5 μM MK801 into the LC, respectively. ** *p* < 0.01; relative to control by MANOVA with Tukey’s post hoc test.

Perfusions with 1 μM MUS into the LC decreased both basal and MK801-induced releases of norepinephrine in the OFC [F_MUS_ (2,15) = 14.3 (*p* < 0.01), F_Time_ (3.6, 35.6) = 106.4 (*p* < 0.01), F_MUS*Time_ (3.6, 35.6) = 7.1 (*p* < 0.01)] and RTN [F_MUS_ (2,15) = 12.2 (*p* < 0.01), F_Time_ (3.5, 35.2) = 42.6 (*p* < 0.01), F_MUS*Time_ (3.5, 35.2) = 2.4 (*p* > 0.05)] (Figure 5A). Perfusions with 1 μM MUS into the LC also decreased both basal and MK801-induced releases of dopamine in the OFC [F_MUS_ (2,15) = 6.0 (*p* < 0.01), F_Time_ (4.9, 49.4) = 37.6 (*p* < 0.01), F_MUS*Time_ (4.9, 49.4) = 1.1 (*p* > 0.05)] (Figure 5B). Taken together with results in Study_1, the results of Study_2 indicate that MK801 generates intra-LC GABAergic disinhibition resulting in the increase in catecholamine release in the OFC and RTN.

### 3.3. Interaction between NMDAR in the LC and Adrenoceptor in the RTN on Transmitter Release in the RTN, and MDTN (Study_3)

To explore the mechanisms of MK801-induced releases of norepinephrine in the RTN and GABA in the MDTN, the effects of perfusion with 100 μM PRZ (α1 adrenoceptor antagonist) and 0.2 μM GUNA (α2A adrenoceptor agonist) into the RTN on MK801-induced releases of catecholamine in the RTN and GABA in the MDTN were determined. Perfusion mediums in the MDTN were maintained with MRS alone during Study_3. Perfusion mediums in the LC was commenced with MRS. Perfusion medium in the RTN was commenced with containing without (control) or with 100 μM PRZ or 0.2 μM GUNA. After the stabilisation of transmitter level in perfusate, perfusion medium in the LC was switched to MRS containing with 5 μM MK801 for 180 min.

#### 3.3.1. Effects of Local Administration of PRZ and GUNA into the RTN on MK801-Induced Catecholamine Release in the RTN

**Figure 6:**
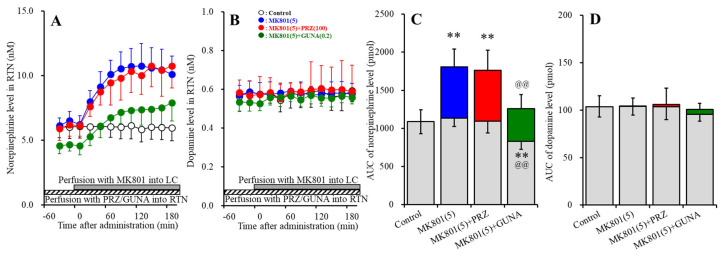
Effects of local administration of PRZ and GUNA into the RTN on MK801-induced releases of norepinephrine (**A**) and dopamine (**B**) in the RTN. Ordinates: mean ± SD (n = 6) of extracellular catecholamine levels (nM); abscissa: time after MK801 administration (min). Stripped bars indicate perfusion with 100 μM PRZ or 0.2 μM GUNA into the RTN. Gray bars indicate perfusion with 5 μM MK801 into the LC. (**C**,**D**) indicate AUC values of extracellular levels of norepinephrine and dopamine (pmol) after perfusion with MK801 (from 20 to 180 min), based on (**A**) and (**B**), respectively. Gray columns represent the AUC values before perfusion with MK801 (basal release). ** *p* < 0.01; relative to control, and @@ *p* < 0.01; relative to MK801 (5 μM) by MANOVA with Tukey’s post hoc test.

Perfusions with MK801 (5 μM) into the LC increased extracellular norepinephrine level in the RTN (MK801-induced release in the RTN) (Figure 6A,C). Perfusion with 100 μM PRZ (α1 adrenoceptor antagonist) into the RTN affected neither basal nor MK801-induced norepinephrine releases in the RTN (Figure 5A,C). Perfusion with 0.2 μM GUNA (α2A adrenoceptor agonist) into the RTN decreased both basal and MK801-induced norepinephrine releases in the RTN [F_GUNA_ (2,15) = 20.8 (*p* < 0.01), F_Time_ (5.2, 78.0) = 68.7 (*p* < 0.01), F_GUNA*Time_ (10.4, 78.0) = 20.8 (*p* < 0.01)] (Figure 6A,C). Perfusions with MK801 (5 μM) into the LC did not affect extracellular dopamine level in the RTN (Figure 6B,D). Neither perfusion with 100 μM PRZ nor 0.2 μM GUNA into the RTN affected dopamine releases in the RTN (Figure 6B,D).

#### 3.3.2. Effects of Local Administration of PRZ and GUNA into the RTN on MK801-Induced GABA Release in the MDTN

**Figure 7:**
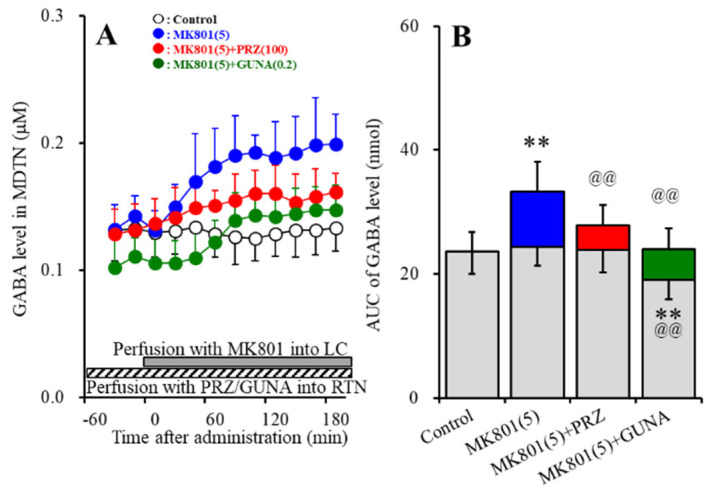
Effects of local administration of PRZ and GUNA into the RTN on MK801-induced GABA release (**A**) in the MDTN. Ordinate: mean ± SD (n = 6) of extracellular GABA level (μM); abscissa: time after MK801 administration (min). Stripped bars indicate perfusion with 100 μM PRZ or 0.2 μM GUNA into the RTN. Gray bars indicate perfusion with 5 μM MK801 into the LC. (**B**) indicates the AUC values of extracellular GABA level (nmol) after perfusion with MK801 (from 20 to 180 min), based on (**A**). Gray columns represent the AUC values before perfusion with MK801 (basal release). ** *p* < 0.01; relative to control, @@ *p* < 0.01; relative to MK801 (5 μM) by MANOVA with Tukey’s post hoc test.

Perfusions with MK801 (5 μM) into the LC increased extracellular GABA level in the MDTN (MK801-induced GABA release in the MDTN) (Figure 7A,B). Perfusion with 100 μM PRZ into the RTN did not affect basal GABA release in the MDTN, but decreased MK801-induced GABA release in the MDTN [F_PRZ_ (2,15) = 8.2 (*p* < 0.01), F_Time_ (3.7, 55.4) = 34.9 (*p* < 0.01), F_PRZ*Time_ (7.4, 55.4) = 7.4 (*p* < 0.01)] (Figure 7A,B). Perfusion with 0.2 μM GUNA into the RTN decreased both basal and MK801-induced GABA release in the MDTN [F_GUNA_ (2,15) = 10.7 (*p* < 0.01), F_Time_ (3.3, 48.7) = 57.3 (*p* < 0.01), F_GUNA*Time_ (6.5, 48.7) = 18.6 (*p* < 0.01)] (Figure 7A,B).

### 3.4. Interaction between NMDAR in the LC and Adrenoceptor in the RTN on AMPA-Evoked Releases of Norepinephrine, Dopamine and L-Glutamate in the OFC (Study_4)

To explore the interaction between thalamocortical glutamatergic transmission and mesocortical catecholaminergic transmission, the effects of perfusion with 100 μM PRZ (α1 adrenoceptor antagonist) or 0.2 μM GUNA (α2A adrenoceptor agonist) into the RTN and perfusion with 5 μM MK801 into the LC on AMPA-induced releases (perfusion with 100 μM AMPA into the MDTN) [29,30,31,33,36,40,41,53] of norepinephrine, dopamine and L-glutamate in the OFC were determined. Perfusion mediums in the OFC was maintained with MRS alone during Study_4. Perfusion mediums in the LC was commenced with MRS containing without (control) or with 5 μM MK801. Perfusion medium in the RTN was commenced with containing without (control) or with 100 μM PRZ or 0.2 μM GUNA. After the stabilisation of transmitter level in perfusate, perfusion medium in the MDTN was switched to MRS containing with 100 μM AMPA for 180 min.

**Figure 8:**
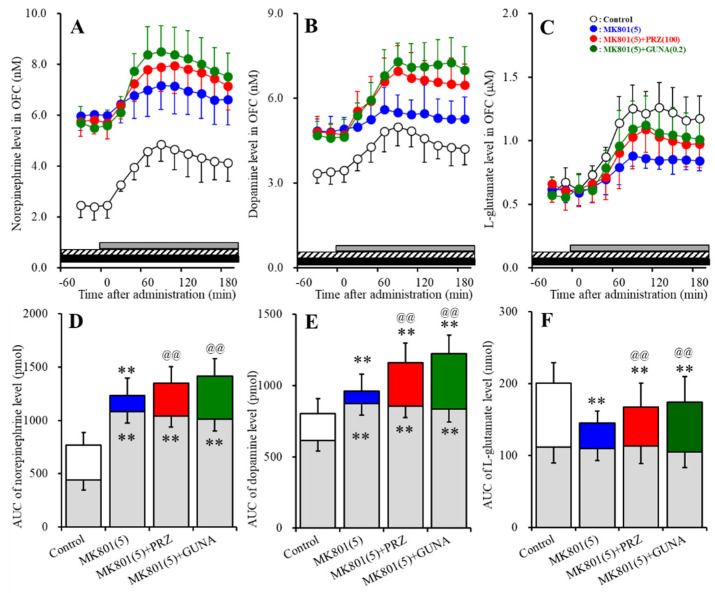
Effects of local administration of MK801 into the LC, PRZ and GUNA into the RTN on α-amino-3-hydroxy-5-methyl-4-isoxazolepropionic acid (AMPA)-induced releases of norepinephrine (**A**,**D**), dopamine (**B**,**E**) and L-glutamate (**C**,**F**) in the OFC. Ordinate: mean ± SD (n = 6) of extracellular levels of norepinephrine (nM), dopamine (nM) and L-glutamate (μM); abscissa: time after AMPA administration (min). Closed and stripped bars indicate perfusion with 5 μM MK801 into the LC, and 100 μM PRZ or 0.2 μM GUNA into the RTN, respectively. Gray bars indicate perfusion with 100 μM AMPA into the MDTN. (**D**–**F**) indicate the AUC values of extracellular levels of norepinephrine (pmol), dopamine (pmol) and L-glutamate (nmol) after perfusion with AMPA (from 20 to 180 min), based on (**A**–**C**), respectively. Gray columns represent the AUC values before perfusion with AMPA (basal release). ** *p* < 0.01; relative to control, @@ *p* < 0.01; relative to MK801 (5 μM) by MANOVA with Tukey’s post hoc test.

Perfusions with 100 μM AMPA into the MDTN increased extracellular levels of norepinephrine, dopamine and L-glutamate in the OFC (AMPA-induced release) (Figure 8A–F). Perfusion with 5 μM MK801 into the LC increased extracellular levels of norepinephrine and dopamine in the OFC (Figure 8A,B,D,E) without affecting that of L-glutamate (Figure 8C,F). Neither perfusion with 100 μM PRZ (α1 adrenoceptor antagonist) nor 0.2 μM GUNA (α2A adrenoceptor agonist) into the RTN affected extracellular levels of norepinephrine, dopamine and L-glutamate in the OFC (Figure 8A–F).

Interaction between perfusion with 5 μM MK801 into the LC and 100 μM PRZ into the RTN on AMPA-induced releases of norepinephrine [F_PRZ_ (2,15) = 28.5 (*p* < 0.01), F_Time_ (9,135) = 71.6 (*p* < 0.01), F_PRZ*Time_ (18,135) = 3.7 (*p* < 0.01)], dopamine [F_PRZ_ (2,15) = 15.2 (*p* < 0.01), F_Time_ (9,135) = 50.3 (*p* < 0.01), F_PRZ*Time_ (18,135) = 2.4 (*p* < 0.01)] and L-glutamate [F_PRZ_ (2,15) = 5.5 (*p* < 0.05), F_Time_ (5.7, 85.0) = 118.9 (*p* < 0.01), F_PRZ*Time_ (11.3, 85.0) = 7.0 (*p* < 0.01)] in the OFC was detected (Figure 8A–F). Interestingly, perfusion with MK801 into the LC reduced AMPA-induced releases of norepinephrine, dopamine and L-glutamate in the OFC (Figure 8A–F). Perfusion with PRZ into the RTN prevented the inhibitory effects of perfusion with MK801 into the LC on AMPA-induced releases of norepinephrine, dopamine and L-glutamate in the OFC (Figure 8A–F).

Interaction between perfusion with 5 μM MK801 into the LC and 0.2 μM GUNA into the RTN on AMPA-induced releases of norepinephrine [F_GUNA_ (2,15) = 31.1 (*p* < 0.01), F_Time_ (6.0, 89.4) = 95.6 (*p* < 0.01), F_GUNA*Time_ (11.9, 89.4) = 8.0 (*p* < 0.01)], dopamine [F_GUNA_ (2,15) = 18.4 (*p* < 0.01), F_Time_ (4.9, 74.1) = 65.3 (*p* < 0.01), F_GUNA*Time_ (9.9, 74.4) = 13.2 (*p* < 0.01)] and L-glutamate [F_GUNA_ (2,15) = 5.2 (*p* < 0.05), F_Time_ (5.4, 80.9) = 159.2 (*p* < 0.01), F_GUNA*Time_ (10.8, 80.9) = 8.5 (*p* < 0.01)] in the OFC was detected (Figure 8A–F). Similar to PRZ, perfusion with GUNA into the RTN prevented the inhibitory effects of perfusion with MK801 into the LC on AMPA-induced releases of norepinephrine, dopamine and L-glutamate in the OFC (Figure 8A–F).

## 4. Discussion

**Figure 9:**
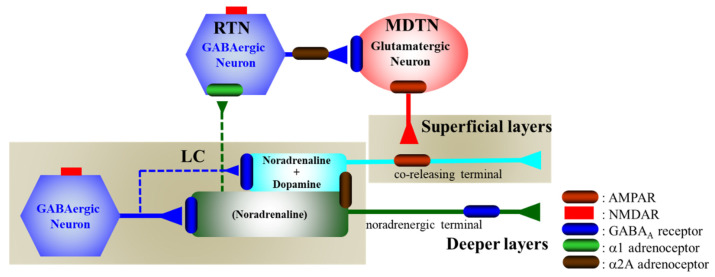
Proposed hypothesis for the extended neural circuitry involved in thalamocortical (RTN–MDTN–OFC) glutamatergic, mesothalamic (LC-MDTN) noradrenergic, and mesocortical (LC-OFC) catecholaminergic connectivity and their regulation systems. GABAergic neurones in the LC receive excitatory glutamatergic inputs via NMDAR. Both noradrenergic and catecholaminergic neurones in the LC are regulated by the inhibitory postsynaptic GABA_A_ receptor and somatodendritic α2A adrenoceptor, but are not affected by the α1 adrenoceptor [36,57,58]. Noradrenergic neurons in the LC project terminals to the OFC (deeper layers) [37,38,56,59] and RTN [35], whereas catecholaminergic neurones in the LC project coreleasing terminal to the OFC (superficial layers) [37,38,56,59], but not to RTN [35]. GABAergic neurones in the RTN receive excitatory noradrenergic input from the LC via α1 adrenoceptor [36]. The intrathalamic GABAergic pathway is regulated by the presynaptic/somatodendritic α2A adrenoceptor [36]. Activated intrathalamic GABAergic transmission suppresses glutamatergic neurones in the MDTN, resulting in the inhibition of thalamocortical (MDTN-OFC) glutamatergic transmission [27,29,35]. The thalamocortical (MDTN-OFC) glutamatergic pathway activates catecholaminergic coreleasing terminals via AMPAR, but does not make contact with the noradrenergic terminals in the OFC [37,38,56,59].

The present study demonstrated the presence of the regulatory systems of the transmission in thalamocortical (MDTN-OFC) glutamatergic, intrathalamic (RTN-MDTN) GABAergic, mesothalamic (LC-RTN) noradrenergic, mesocortical (LC-OFC) noradrenergic and catecholaminergic (norepinephrine/dopamine coreleasing) pathways, using multiprobes microdialysis. According to the results in this study and the published neural circuits [26,27,30,31,36,37,38,54,55,56], our proposed hypothesis regarding the neural networks from the LC associated with NMDAR are indicated in Figure 9.

### 4.1. Catecholaminergic Transmission Regulating System Associated with NMDAR

The inhibition of NMDAR in the medial prefrontal cortex, insula, and dorsal raphe nucleus (DRN) increased regional monoamine release via GABAergic disinhibition [30,31,34,37,38,43,48,52]. The noradrenergic and catecholaminergic neurones in the LC project three pathways; namely, the mesocortical noradrenergic and catecholaminergic coreleasing (dopamine with norepinephrine) pathways, as well as the mesothalamic (LC-RTN) noradrenergic pathway [36] (Figure 9). The present study also demonstrated that the noradrenergic neurones in the LC project a selective noradrenergic terminal to both OFC and RTN, but catecholaminergic neurones in the LC project coreleasing terminal to the OFC but not to the RTN. Contrary to catecholamine, the local administration of MK801 into the LC decreased regional GABA release. Therefore, these increases in releases of norepinephrine and dopamine induced by the local administration of MK801 (MK801-induced release) were generated by intra-LC GABAergic disinhibition, as the local administration of MUS (GABA_A_ receptor agonist) prevented the MK801-induced releases of norepinephrine and dopamine in the OFC and RTN. Therefore, MK801 inhibits the GABAergic neuronal activity without affecting the noradrenergic and catecholaminergic neurones in the LC. Similar to the LC, we have already demonstrated the MK801-induced enhancement of monoaminergic transmission in the frontal cortex [37,38,48,52], serotonergic transmission in the DRN [30,31,34], and glutamatergic transmission in the thalamus [27,28,29,33,40] via regional GABAergic disinhibition.

During the resting stage, cation channels in NMDAR are inhibited by Mg^2+^ and Zn^2+^ caps via specific binding sites; however, depolarization (higher than −20 mV) repels Mg^2+^ and Zn^2+^ from the channel pore, leading to the voltage-dependent inflow of Na^+^ and Ca^2+^, and outflow K^+^ [60]. Therefore, NMDAR is a ligand-gated cation channel with accompanying voltage-sensitive features. Additionally, the resting membrane potential of GABAergic interneurons is usually positive (−50 ~ −60 mV) compared with that of monoaminergic and glutamatergic neurons [61,62]. Therefore, the unique profiles of the selective inhibitory effects of MK801 on GABAergic transmission rather than monoaminergic or glutamatergic transmissions are induced by the differences of the resting membrane potential between the GABA neurones and other neurones.

The threshold concentration of the local administration of MK801 on the release of norepinephrine and dopamine in the OFC were lower than 1 μM and 5 μM, respectively. Therefore, the selective noradrenergic projection is predominantly sensitive to NMDAR, rather than that of catecholaminergic projection. Furthermore, the noradrenergic projection to the OFC is more sensitive to NMDAR rather than that to the RTN, as the threshold concentration of MK801 on norepinephrine release in the RTN was lower than 5 μM. These distinct sensitivities between noradrenergic and catecholaminergic projections constitute the unique regulation system of catecholaminergic transmission in the OFC. In LC-OFC pathways, noradrenergic and catecholaminergic neurones project deeper and superficial layers in the OFC, respectively [36,37,38,56,59]. The thalamocortical glutamatergic pathway projects to the superficial layers in the frontal cortex [35,37,38]. Therefore, the thalamocortical glutamatergic pathway makes contact with the catecholaminergic coreleasing terminal, but not with noradrenergic terminal in the OFC [36,37,38]. The MK801-induced norepinephrine release in the RTN inhibited thalamocortical (MDTN-OFC) glutamatergic transmission via the activation of intrathalamic GABAergic inhibition, as local administration in the LC unexpectedly increased GABA release in the MDTN. These results suggest that the functional abnormalities induced by NMDAR inhibition in the LC affect various transmission systems, and may be more complex than our expectation. Indeed, intra-LC GABAergic disinhibition by NMDAR inhibition in the LC increased the release of norepinephrine and dopamine in the OFC via the activation of both noradrenergic and catecholaminergic neurones in the LC, but enhanced noradrenergic transmission in the RTN leads to the suppression of the thalamocortical catecholaminergic coreleasing terminal via the inactivation of the thalamocortical glutamatergic transmission in the OFC.

### 4.2. Catecholaminergic Transmission Regulating System Associated with Adrenoceptor

Cortical α2A adrenoceptor is predominantly expressed in the postsynaptic spine, whereas the α2A adrenoceptor in the LC are expressed in both the presynaptic and somatodendritic regions, and function as autoreceptors on both non-catecholaminergic and catecholaminergic terminals [57]. Contrary to the α2A adrenoceptor, the α1 adrenoceptor has restricted expression regions compared with the α2 receptors, and is expressed in the thalamus rather than that in the LC [58] (Figure 9). Noradrenergic neurons in the LC directly project to the RTN and enhance GABAergic transmission via the activation of postsynaptic α1 adrenoceptor [36,63].

In the present study, local administrations of PRZ (α1 adrenoceptor antagonist) into the LC and RTN did not affect basal or MK801-induced norepinephrine release in the OFC and RTN; however, GABA release in the MDTN induced by the local administration of MK801 in the LC was inhibited by the local administration of PRZ into the RTN, but not by that into the LC. Therefore, elevation of norepinephrine release into the RTN enhances GABAergic neurones in the RTN via the activation of the postsynaptic α1 adrenoceptor. Contrary to the α1 adrenoceptor, local administrations of GUNA (α2A adrenoceptor agonist) into the LC decreased the basal and MK801-induced norepinephrine release in the OFC and RTN. Furthermore, local administrations of GUNA into the RTN also decreased basal and MK801-induced norepinephrine release in the RTN. Therefore, the activation of somatodendritic α2A in the LC and presynaptic α2A adrenoceptor in the RTN inhibits mesothalamic noradrenergic transmission. The paradoxical activation of intrathalamic GABAergic inhibition induced by local administration in the LC was prevented by the activation of the somatodendritic α2A adrenoceptor in the LC, presynaptic α2A adrenoceptor in the RTN, and/or inhibition of postsynaptic α1 adrenoceptor in the RTN. In other words, the modulation of these adrenoceptor subtypes can compensate the suppression of the thalamocortical glutamatergic transmission induced by the inhibition of NMDAR in the LC.

### 4.3. Clinical Implication of NMDAR Antagonist and Adrenoceptor Agents

The functional magnetic resonance study demonstrated that the subanaesthetic dose of ketamine generated the reduced functional connectivity between the LC and MDTN [42]. This study provided the mechanisms of ketamine-induced reduced connectivity between the LC and MDTN. Enhanced intrathalamic (RTN-MDTN) GABAergic inhibition induced by the hyperactivation of mesothalamic (LC-RTN) noradrenergic transmission inhibits the neuronal activity of glutamate neurons in the MDTN. Therefore, the reduced functional connectivity between LC and MDTN induced by ketamine is possibly due to the observed suppression of MDTN neuronal activity via the hyperactivation of intrathalamic GABAergic inhibition.

Regarding the attention mechanism, the physiological enhancement of the thalamic activity generates a reduction in distraction in the attentional performance [64]; however, the pathological enhancement of the thalamocortical glutamatergic transmission induced by phencyclidine generates a working memory deficit, but is compensated by a therapeutic-relevant dose of GUNA [65]. Therefore, the imbalance between the neuronal hyperactivation in the LC and the hypofunction in the MDTN are related to a change in the regulation of arousal in terms of a disruption of the alerting function, a promotion of non-selective sensory signal detection, and an enhancement of behavioural flexibility [42].

Contrary to thalamocortical glutamatergic transmission, the enhancement of the postsynaptic α2A adrenoceptor in the frontal cortex improves cognitive deficits, including working memory, but activations both frontal α1 and β adrenoceptors, which impair working memory [66]. Indeed, the relatively lower extracellular norepinephrine level during pre-event periods with event-related transient increased norepinephrine release in OFC contributes to generating stability and flexibility against contingency and uncontrolled severe stresses [36]. Therefore, the persistent relatively lower noradrenergic tone in OFC generates stable and sticks in an initial state, but discards a critical change, whereas the continuous hyper-noradrenergic tone in the OFC reliably leads unstable and cycles concentrations to a sequence of environmental changes [36,67]. Therefore, an appropriate dose (relatively low dose) NMDAR antagonist possibly has the potential for improving working memory/cognition via the selective enhancement of mesocortical noradrenergic transmission without affecting mesothalamic noradrenergic transmission, as the threshold concentration of the local administration of MK801 into the LC on mesocortical selective noradrenergic transmission was lower than 1 μM, but those on the mesothalamic noradrenergic transmissions were lower than 5 μM.

Further complex considerations are required regarding the relationship between mesocortical catecholaminergic coreleasing transmission and cognition. In our previous studies, we demonstrated that both local (into the RTN) and systemic administrations of MK801 enhanced the thalamocortical glutamatergic transmission concentration dose-dependently [28,29,35]; however, the MK801-induced activation of thalamocortical glutamatergic transmission is compensated by antipsychotics, clozapine (activation of group III metabotropic glutamate receptor), aripiprazole (activation of group II metabotropic glutamate receptor), and lurasidone (inhibition of serotonin 5-HT7 receptor) [26,27,30,31]. The incomprehensible results regarding the suppression of thalamocortical glutamatergic transmission induced by the local administration of the NMDAR antagonist, which aggravates or conducts psychosis and cognitive deficits, were similar to the actions of antipsychotics. We speculate that the paradoxical mechanisms that direct the inhibition of NMDAR are possibly predominant compared with the postsynaptic α1 adrenoceptor secondarily activation in the RTN GABAergic neurones, based on the systemic MK801-induced dose-dependent activation of thalamocortical glutamatergic transmission [26,28,29]. Therefore, the systemic administration of the NMDAR antagonist double-burstly enhances mesocortical catecholaminergic coreleasing transmission. Although this hypothesis needs to be clarified in further study, a relatively low dose NMDAR antagonist can selectively enhance mesocortical noradrenergic transmission, without affecting mesocortical catecholaminergic coreleasing transmission, whereas conversely, a relatively high-dose NMDAR antagonist enhances both mesocortical selective noradrenergic and catecholaminergic coreleasing transmissions in the OFC.

GUNA, which is approved for the treatment of ADHD, enhances thalamocortical glutamatergic transmission via the inhibition of mesothalamic (LC-RTN) noradrenergic transmission [36]. In the frontal cortex, including the medial prefrontal cortex, insula, and OFC, the thalamocortical glutamatergic terminal enhances the mesocortical catecholaminergic coreleasing terminal from the LC [37,38]. Therefore, the impacts of the thalamocortical glutamatergic and mesocortical catecholaminergic transmission on cognitive function might be synonymous in the neuronal networks from the LC. Contrary to noradrenergic transmission, the inhibition of NMDAR in the DRN activates thalamocortical glutamatergic transmission via the activation of the serotonin 5-HT7 receptor in the MDTN [30,31,34]. The contradictive actions between the serotonergic and noradrenergic transmissions on the thalamocortical glutamatergic transmission possibly, at least partially, can explain the clinical advantage of selective serotonin transporter inhibitors to major depression, compared with selective norepinephrine transporter inhibitors. Both serotonergic and noradrenergic transmissions are recognized as fundamental transmission systems in the pathophysiology of major depression, whereas noradrenergic compounds have been less extensively utilized in clinical medication against major depression compared with selective serotonin transporter inhibitors. The development of the selective norepinephrine transporter inhibitors, reboxetine and atomoxetine, have not substantially improved the state of the art against mood disorders medication [68]. Additionally, the antidepressive mechanisms of atypical antipsychotics, quetiapine/norquetiapine, approved for the treatment of bipolar depression, are mediated by their α1 adrenoceptor inhibition [38,69]. Taken together with clinical evidence, the present results suggest that noradrenergic transmission is likely not sufficient to obtain a full antidepressive action, but the suppression of mesothalamic noradrenergic transmission via α1 adrenoceptor inhibition and/or α2A adrenoceptor activation represents an important element for effectiveness in the treatment of depressive or bipolar disorder with anxious distress/mixed features.

## 5. Conclusions

The present study, to clarify the effects of NMDAR in the LC on thalamocortical (RTN-MDTN-OFC) GABAergic/glutamatergic, mesothalamic (LC-RTN) noradrenergic, and mesocortical (LC-OFC) selective noradrenergic and coreleasing catecholaminergic transmissions, determined the effects of the local administration of noncompetitive NMDAR antagonist, MK801, into the LC on releases of L-glutamate, GABA, norepinephrine, and dopamine in the OFC, RTN, MDTN, and LC, using multiprobe microdialysis. The inhibition of NMDAR in the LC generates intra-LC GABAergic disinhibition, resulting in enhancement in mesocortical selective noradrenergic and catecholaminergic coreleasing (norepinephrine and dopamine) transmissions and mesothalamic noradrenergic transmission. Interestingly, thalamocortical glutamatergic transmission is suppressed by the enhanced intrathalamic (RTN-MDTN) GABAergic inhibition, due to the MK801-induced activation of mesothalamic noradrenergic transmission. Intrathalamic (RTN-MDTN) GABAergic transmission was inhibited by the inhibition of the postsynaptic α1 adrenoceptor in the RTN, and the activations of the somatodendritic α2A adrenoceptor in the LC or presynaptic α2A adrenoceptor in the RTN. Remarkably, the sensitivity of mesocortical selective noradrenergic transmission to MK801 is relatively higher than those of mesothalamic noradrenergic and mesocortical catecholaminergic transmissions. Therefore, a relatively low dose of NMDAR antagonist possibly enhances mesocortical noradrenergic transmission without affecting mesocortical catecholaminergic or mesothalamic noradrenergic transmissions; however, a relatively high dose of NMDAR antagonist enhances both mesothalamic noradrenergic, mesocortical noradrenergic, and catecholaminergic transmissions. These distinct sensitivities of the three pathways associated with the LC probably contribute to the pathophysiology of cognitive deficits and mood disorders associated with NMDAR.
